# Effect of Processing and Cooking on Physicochemical, Sensory, and Functional Properties of Food

**DOI:** 10.3390/foods14091598

**Published:** 2025-05-01

**Authors:** Sheng-Dun Lin

**Affiliations:** Department of Food Science and Technology, Hungkuang University, 1018, Sec. 6, Taiwan Boulevard, Shalu District, Taichung 433304, Taiwan; lin54@hk.edu.tw; Tel.: +886-4-26318652 (ext. 5038); Fax: +886-4-26319176

Food ingredients come from a wide range of sources, and different processing and cooking methods can change their appearance, nutrients and phytochemicals, thereby increasing the product’s shelf life, palatability and functionality. In addition, the reuse of natural resources can be improved through innovative processing or cooking techniques. The main focus of this Special Issue is the “Effect of Processing and Cooking on Physicochemical, Sensory, and Functional Properties of Food”, which is linked to the maintenance or enhancement of the nutritional quality, bioactive components and functionality of food through processing or cooking techniques or product formulation. This Special Issue contains 16 high-quality original research articles on enhancing the nutritional value and functionality of traditional foods, the reuse of processing by-products, the effects of different processing and cooking methods on food quality characteristics, and the application of new food processing technologies.

## 1. Enhancing the Nutritional Value and Functionality of Traditional Foods

Many research reports have pointed out that plant foods, in addition to providing essential nutrients, are also rich in a variety of bioactive compounds [1,2]. These bioactive compounds are directly related to human health and various conditions [3,4], such as cardiovascular disease and metabolic syndrome. Therefore, if foods containing bioactive ingredients can be added to traditional foods using processing technology, their nutritional value, antioxidant properties, and health benefits can be enhanced [4,5]. Traditional wheat products, such as wheat noodles, baked bread and steamed bread, are popular among consumers, but are often criticized for lacking bioactive compounds and dietary fiber.

The flowers of *Clitoria ternata* (CT) can be used as a natural pigment in the food industry. The flowers and extracts of CT are rich in bioactive compounds and possess antioxidant, anti-inflammatory, antibacterial, antidiabetic and antitumor activities [6]. Shiau et al. (contribution 1) investigated the effects of CT flower extract (CTFE) on the quality characteristics of dry and boiled noodles. The results showed that cooking the noodles significantly decreased their anthocyanin content and blue color, but increased the green intensity of the noodles. The addition of 20–30% CTFE produced blue noodles with rich bioactive substances, antioxidant and good sensory attributes compared to the control.

Tamarillo (*Solanum betaceum* Cav.) has a unique flavor and is rich in nutrients, bioactive compounds, and health benefits [4,7]. Syu et al. (contribution 3) investigated the effects of freeze-dried tamarillo powder (TP) on the physicochemical, sensory, and functional properties of mantou. The results showed that TP was rich in phytochemicals and had good antioxidant properties. Taking wheat bread (a high GI food) as a reference, T20 (manto made by replacing 20% of wheat flour with TP) was classified as a medium GI food. Adding an additional 15–20% water to the T20 formulation increased its volume and specific volume, and the overall liking score (7.4–7.5 on a 9-point hedonic test) was not significantly different from the control (*p* > 0.05). A new medium GI manto with more bioactive compounds and good antioxidant properties was successfully developed.

Frozen dough is increasingly used in bread and bakery products, but the freezing and thawing process during production destroys the gluten structure and prevents the bread from expanding, resulting in a smaller volume and a tougher crumb [8]. Casein hydrolysate can not only be used as a protein supplement, but can also improve the quality characteristics of various foods [9]. The effects of casein savinase hydrolysate (CSH) (1–2 g/100 g flour) on the physicochemical quality properties of wheat dough and corresponding bread were studied by Bekiroglu et al. (contribution 9). This study confirmed that adding CSH to frozen dough can reduce the quality decline of frozen bread.

## 2. Reuse of Processing By-Products

Nutrient-rich and safe agricultural processing by-products can be sustainably used to produce value-added products with economic and environmental benefits [10]. Cocoa bean shells (CBSs) are a by-product of chocolate processing (accounting for approximately 12–20% of cocoa beans) and their nutritional composition is not significantly different from cocoa beans and should not be discarded [11]. Wang et al. (contribution 16) investigated the effects of replacing 5–25% of wheat flour (WF) with Taiwanese cocoa bean shells (CBSs) on the quality properties of bread. The heavy metal content of CBS is lower than the Codex Alimentarius limit for cocoa powder, and ochratoxin A and aflatoxin (B1, B2, G1 and G2) were not detected. CBS is not only rich in nutrients but also has good antioxidant properties. This study also successfully developed a new medium GI bread containing total dietary fiber and phytochemicals by partially replacing wheat flour with CBS.

During the process of making mushrooms into vacuum fried crisps, the blanching broth (BB) and centrifuged broth (CB) generated are often discarded, increasing the amount of wastewater and treatment costs. Chen et al. (contribution 5) determined the quality characteristics of BB and CB and formulated them into instant beverages using functional indigestible dextrin. The BB and CB are rich in bioactive compounds, especially polysaccharides, ergothioneine, and total phenols. This study successfully developed BB and CB into functional foods in the form of beverages.

## 3. Effects of Different Processing and Cooking Methods on Food Quality Characteristics

Appropriate processing or cooking techniques are an important research topic because they can maintain or improve the nutrients, phytochemicals, sensory and functional properties of foods. Glucosinolates can be hydrolyzed into isothiocyanates, antithyroid substances, and goitrogens. Research has shown that washing, soaking, and boiling (destroy myrosinase) can help reduce the amount of goitrogens in food and prevent the release of goitrogens from thyrotropin [12]. However, heating also destroys glucosinolates and isothiocyanates [13]. Panduang et al. (contribution 7) investigated the optimization of cooking conditions to reduce goitrogens while retaining isothiocyanates in cruciferous vegetables. The results showed that home cooking methods to preserve isothiocyanates and reduce goitrogens include steaming cabbage at 80–100 °C for 4 min and stir-frying Chinese kale at 60–100 °C for 2 min. These methods can preserve phytochemicals and reduce food hazards.

Vegetables can be pickled through anaerobic fermentation with lactic acid bacteria, acetic acidification with or without added salt, or a combination of these processes [14]. Radman et al. (contribution 8) prepared unfermented sea fennel leaf preservatives using different pickle juices made from apple cider vinegar, wine vinegar, and alcohol vinegar and compared their quality properties. The results showed that sea fennel pickled in alcohol vinegar scored the highest in all sensory parameters, while sea fennel samples pickled in wine vinegar scored the lowest. A strong odor of wine vinegar was described as an off-flavor of this sample.

Parboiling and polishing are common rice processing methods in Bangladesh. Akhter et al. (contribution 10) studied the effects of parboiling and polishing on the nutritional composition of five high-yielding rice varieties. The results suggest that consumers should be encouraged to consume unpolished rice in order to reduce the loss of nutrients in rice.

The cooking process can significantly change the quality characteristics of meat. Wang et al. (contribution 11) studied the effects of different frying temperatures and times on the physicochemical properties and volatile components of pork tenderloin, and their correlations. The results show that b* value and moisture content have the greatest impact on volatile substances. The b* value was positively correlated with the aroma components, but negatively correlated with the moisture content. This study suggests that the b* value and moisture content can be detected to predict the changes in the types and contents of volatile substances in fried pork.

Frankfurters, bratwursts, and wieners are becoming less popular among consumers due to their lack of fiber or high salt, nitrite, and fat content [15]. Pérez et al. (contribution 12) evaluated the nutritional quality of artisanal sausages produced in 10 factories in Ecuador. The results showed that the proximate composition contents of handmade sausages were within the recommended range for commercial sausages. This suggests that “artisan” is just a name for a product.

Peach palm fruit contains a large amount of oil and protein, as well as a large amount of carbohydrates and insoluble dietary fiber, and is therefore considered to be a fruit with high nutritional value. However, peach palm fruits need to be cooked in water before consumption to inhibit anti-nutritional factors, eliminate oxalate crystals in the peel [16], and inactivate peroxidase in the pulp to avoid irritation of the throat mucosa [17]. Soares et al. (contribution 13) first reported the changes in the quality characteristics of albino peach palm before and after the cooking and drying processes. The results showed that the albino peach palm pulp and powder could be utilized by the food, pharmaceutical and biotechnology industries.

*Agaricus bisporus* has been extensively studied due to its unique quality properties, such as flavor and high amino acid score [18]. However, there are few studies on the changes in its flavor compounds during cooking. Xie et al. (contributor 14) used gas chromatography–mass spectrometry and electronic nose to study the effects of different cooking methods on the volatile aroma components of mushrooms. The results showed that the electronic nose could effectively distinguish samples prepared by different cooking methods. Cluster analysis of differential aroma substances showed that the aroma of the steamed group and the raw group was more similar. 1-Octen-3-one was identified as the main aroma component of cooked mushrooms.

## 4. Application of New Food Processing Technologies

In recent years, many scholars have applied new food processing technologies to product development and quality research. The processing technology of forming three-dimensional structures by stacking edible materials is called three-dimensional food printing (3DFP) technology [19]. The fruits of *Aronia melanocarpa* (AM) are rich in polyphenols with functional properties such as antioxidant, lipid-lowering, anti-inflammatory, and anti-cancer effects [20]. Zhou et al. (contributor 2) aimed to investigate the printability and storage properties of AM gels in 3DFP. The results showed that the AM polyphenol gel loading system most suitable for 3DFP processing and printing requirements was AM slurry:methyl cellulose:pea albumin:hyaluronic acid = 100:14:1:1. This study provides a theoretical basis for 3D printing applications using fruit pulp as raw material.

Ultrasound is a non-thermal radiation technology. Its interaction with the medium will produce mechanical effects, cavitation effects and thermal effects [21]. Ultrasonic pretreatment can effectively enhance heat transfer, improve drying efficiency and inhibit the deterioration of material quality. Shang et al. (contributor 4) studied the effects of different ultrasonic pretreatment processes on the quality properties of licorice. Compared with the control, ultrasonic pretreatment combined with far-infrared drying significantly shortened the drying time of the samples, reduced the moisture content, and improved the quality of the samples. The results of this study can be used as a reference for the industrialization of licorice drying.

The traditional frying drying method is to heat the oil to 160–180 °C under atmospheric pressure to dry out the moisture in the raw materials through boiling and evaporation in contact with air [22]. However, the physical and chemical properties of food will change during the frying process, causing defects such as rancid odor, dark brown color, and shrinkage in shape. Compared with traditional frying, vacuum frying is a process in which the frying and heating process is carried out in a vacuum and sealed container [23]. Since the boiling point of water decreases under reduced pressure, the water in the vacuum system evaporates quickly and is removed even at low oil temperatures. The resulting dehydrated food becomes crispy while retaining its original color and flavor. Vacuum frying is rarely used in the production of aquatic products (especially fish). Chien et al. (contributor 6) studied the application of vacuum frying technology in the preparation of dried silver herring products. The results showed that this technology was suitable for preparing dried herring products. Vacuum frying temperature significantly affects the water activity, oil content and color of the samples. This study can provide seafood processors with optimal processing conditions to achieve better quality.

Cold plasma (CP) technology can reduce the use of chemicals, so its introduction into the food industry is of great significance [24]. Plasma-reactive substances can reduce microbial contamination, modify specific food structures, and extract nutrients from samples [25]. Cao et al. (contributor 15) evaluated the quality characteristics of rice grains treated repeatedly with low-pressure radio frequency (RF) helium CP. The results showed that low-pressure RF 120W helium CP treatment for 20 s had a lasting effect on rice grains, and intermittent CP treatment had the additional benefit of improving the cooking and gelatinization properties of rice and maintaining the hygroscopic properties of rice.

In conclusion, this Special Issue provides some essential data on the effects of food processing and cooking techniques on food nutritional content, phytochemicals, sensory qualities, antioxidant properties and functionality. It also provides several effective strategies for the innovation and improvement of various types of food in the future, which is of great significance to researchers and food manufacturers. The papers included in this Special Issue have made positive contributions to the research progress in the field of food processing and cooking technology.

## References

[1] Guo Y., Zhang H., Shao S., Sun S., Yang D., Lv S. (2022). Anthocyanin: A review of plant sources, extraction, stability, content determination and modifications. Int. J. Food Sci. Technol..

[2] Nolden A.A., Forde C.G. (2023). The nutritional quality of plant-based foods. Sustainability.

[3] Hemler E.C., Hu F.B. (2019). Plant-based diets for cardiovascular disease prevention: All plant foods are not created equal. Curr. Atheroscler. Rep..

[4] Diep T.T., Rush E.C., Yoo M.J.Y. (2022). Tamarillo (*Solanum betaceum* Cav.): A review of physicochemical and bioactive properties and potential applications. Food Rev. Int..

[5] McClements D.J., Grossmann L. (2024). Next-generation plant-based foods: Challenges and opportunities. Annu. Rev. Food Sci. Technol..

[6] Li C., Tang W., Chen S., He J., Li X., Zhu X., Li H., Peng Y. (2022). Phytochemical Properties and In Vitro Biological Activities of Phenolic Compounds from Flower of *Clitoria ternatea* L.. Molecules.

[7] Chen S.-Y., Zhang Q.-F., Lin S.-D. (2024). Nutritional and phytochemical composition of the red tamarillo grown in Taiwan. J. Food Compos. Anal..

[8] Naito S., Fukami S., Mizokami Y., Ishida N., Takano H., Koizumi M., Kano H. (2004). Effect of freeze-thaw cycles on the gluten fibrils and crumb grain structures of breads made from frozen doughs. Cereal Chem..

[9] Bekiroglu H., Bozkurt F., Karadag A., Ahhmed A.M., Sagdic O. (2022). The effects of different protease treatments on the technofunctional, structural, and bioactive properties of bovine casein. Prep. Biochem. Biotechnol..

[10] Galali Y., Omar Z.A., Sajadi S.M. (2020). Biologically active components in by-products of food processing. Food Sci. Nutr..

[11] Rojo-Poveda O., Barbosa-Pereira L., Zeppa G., Stévigny C. (2020). Cocoa bean shell—A by-product with nutritional properties and biofunctional potential. Nutrients.

[12] Oloyede O.O., Wagstaff C., Methven L. (2021). The Impact of Domestic Cooking Methods on Myrosinase Stability, Glucosinolates and Their Hydrolysis Products in Different Cabbage (*Brassica oleracea*) Accessions. Foods.

[13] Baenas N., Marhuenda J., García-Viguera C., Zafrilla P., Moreno D.A. (2019). Influence of Cooking Methods on Glucosinolates and Isothiocyanates Content in Novel Cruciferous Foods. Foods.

[14] Pérez-Díaz I.M., Breidt F., Buescher R.W., Arroyo-López F.N., Jiménez-Díaz R., Garrido-Fernández A., Bautista-Gallego J., Yoon S.S., Johanningsmeier S.D. (2015). Fermented and Acidified Vegetables. Compendium of Methods for the Microbiological Examination of Foods.

[15] Méndez-Zamora G., García-Macías J.A., Santellano-Estrada E., Chávez-Martínez A., Durán-Meléndez L.A., Silva-Vázquez R., Quintero-Ramos A. (2015). Fat reduction in the formulation of frankfurter sausages using inulin and pectin. Food Sci. Technol..

[16] Pinheiro R.C., Ballesteros L.F., Cerqueira M.A., Rodrigues A.M.C., Teixeira J.A., Silva L.H.M. (2022). Peach palm (*Bactris gasipaes* Kunth) and mammee apple (*Mammea americana* L.) seeds: Properties and potential of application in industry. LWT.

[17] dos Santos M.A.S., Protázio D.C., da Costa G.P., Rebello F.K., Martins C.M., Bezerra A.S., da Silva Nogueira A. (2021). Profile of peach palm fruit consumers in the metropolitan region of Belém, Pará, Brazilian Amazon. Int. J. Innov. Educ. Res..

[18] Krishnamoorthi R., Srinivash M., Mahalingam P.U., Malaikozhundan B. (2022). Dietary nutrients in edible mushroom, *Agaricus bisporus* and their radical scavenging, antibacterial, and antifungal effects. Process Biochem..

[19] Montoya J., Medina J., Molina A., Gutiérrez J., Rodríguez B., Marín R. (2021). Impact of viscoelastic and structural properties from starch-mango and starch-arabinoxylans hydrocolloids in 3D food printing. Addit. Manuf..

[20] Sun W.T., Wang S., Wang M., Xu H.T., Zhou Q.C. (2019). Research Progress of Extraction, Purification and Function of Overseas *Aronia melanocarpa* in Recent Years. Food Ind..

[21] Yuan L., He X., Lin R., Cheng S. (2021). Effect of ultrasonic pretreatment on moisture state and hot air drying characteristics of kiwifruit. J. Agric. Eng..

[22] Sharanabasava M.R. (2018). Vacuum processing of food—A mini review. MOJ Food Process. Technol..

[23] Diamante L.M., Shi S., Hellmann A., Busch J. (2015). Vacuum frying foods: Products, process and optimization. Int. Food Res. J..

[24] Zadeh J.H., Pazir F. (2023). Invesigation of the potential application of cold plasma technology in food safety. GIDA.

[25] Warne G.R., Williams P.M., Pho H.Q., Tran N.N., Hessel V., Fisk I.D. (2021). Impact of cold plasma on the biomolecules and organoleptic properties of foods: A review. J. Food Sci..

